# Timing of Intervention Affects Brain Electrical Activity in Children Exposed to Severe Psychosocial Neglect

**DOI:** 10.1371/journal.pone.0011415

**Published:** 2010-07-01

**Authors:** Ross E. Vanderwert, Peter J. Marshall, Charles A. Nelson, Charles H. Zeanah, Nathan A. Fox

**Affiliations:** 1 Department of Human Development, University of Maryland, College Park, Maryland, United States of America; 2 Department of Psychology, Temple University, Philadelphia, Pennsylvania, United States of America; 3 Division of Developmental Medicine, Children's Hospital Boston and Harvard Medical School, Boston, Massachusetts, United States of America; 4 Department of Psychiatry and Neurology, Tulane University, New Orleans, Louisiana, United States of America; Università di Parma, Italy

## Abstract

**Background:**

Early psychosocial deprivation has profound effects on brain activity in the young child. Previous reports have shown increased power in slow frequencies of the electroencephalogram (EEG), primarily in the theta band, and decreased power in higher alpha and beta band frequencies in infants and children who have experienced institutional care.

**Methodology/Principal Findings:**

We assessed the consequences of removing infants from institutions and placing them into a foster care intervention on brain electrical activity when children were 8 years of age. We found the intervention was successful for increasing high frequency EEG alpha power, with effects being most pronounced for children placed into foster care before 24 months of age.

**Conclusions/Significance:**

The dependence on age of placement for the effects observed on high frequency EEG alpha power suggests a sensitive period after which brain activity in the face of severe psychosocial deprivation is less amenable to recovery.

## Introduction

Institutionalization of children has profound consequences for brain development and functioning [1]–[5]. Previous imaging work has reported reduced glucose metabolism, impaired cortico-cortical connections, and decreased cerebellar volume in children adopted from institutions [2]–[4]. Here, we report on brain electrical activity from the electroencephalogram (EEG) acquired in 8-year-old children who were part of a follow-up study in the Bucharest Early Intervention Project (BEIP). The BEIP is a randomized control trial of foster care as an alternative to institutionalization, which aims to understand the effects of early psychosocial deprivation on behavioral and brain development (for design details see ref [6]).

In the baseline assessment of brain activity from the BEIP study (prior to random assignment), we recorded EEG from institutionalized children while they attended to a benign visual stimulus. We reported that children living in institutions had greater EEG power in the theta band and reduced EEG power in alpha and beta bands when compared to a sample of never-institutionalized children [1]. The profile of brain activity in the children living in institutions was similar to those reported in studies examining EEG power in economically impoverished children, children diagnosed with ADHD, and adults who experienced intrauterine stress [7]–[11]. The patterns of elevated power in low frequency and decreased power in high-frequency EEG observed in these studies have been interpreted as representing a maturational lag in the development of the EEG [1], [7]–[10]. Studies examining the development of the EEG report simultaneous decreasing low frequency power with increasing high frequency power (for a review see ref [12]). Furthermore, activity in higher frequencies like alpha and beta have long been associated with improved cognitive performance, attention state, and arousal in human infants, children, and adults [13]–[16].

Following the baseline assessment, institutionalized children in the BEIP were randomly assigned to remain in the institution and receive care-as-usual or be placed into a foster care program implemented by the research team. When children were 42 months of age, EEG was again collected from children in the care as usual group (CAUG) and foster care group (FCG). After receiving an average of 18 months of foster care, the FCG did not differ from the CAUG children in any of the EEG frequency bands [5].

Our aim here was to further explore the status of brain activity in the FCG as a result of another 4.5 years of foster care intervention. Follow-up assessments, including cognitive, social, and electrophysiological data collection, were completed when the children in the BEIP were 8-years-old and transitioning to school. We hypothesized that, by the time children were 8 years of age and had experienced an average of 6.5 years of environmental enrichment through foster care, EEG power in theta (4–6Hz), alpha (7–12Hz), and beta (13–20Hz) frequency bands in FCG children would be comparable to a comparison sample of never-institutionalized (NIG) Romanian children. To examine these hypotheses, we recorded EEG from 143 children; of those, 48 (25 male) belonged to the CAUG, 53 (28 males) belonged to the FCG, and 42 (25 male) comprised the NIG.

## Results

### Intent-to-Treat

Within the intervening years between assessments, there were changes in the living arrangements for a number of children within the CAUG and FCG (e.g. see ref [6]); for the following analyses, we employed an intent-to-treat approach, whereby the data were treated as if each child had remained within their assigned groups. Therefore, we interpret the current findings as a conservative estimate of the intervention effect.

To assess the effects of the foster care intervention on EEG power, we first compared the CAUG and FCG on the three frequency bands. There were no main effects of group for the theta frequency band, however there was a significant region×hemisphere×group (Greenhouse-Geisser corrected *F*_1.73,170.75_ = 4.634, η^2^ = .045, *p* = .015). Follow-up comparisons revealed that the CAUG had greater theta power at the temporal electrode in the left hemisphere (T7). Analyses for the alpha band revealed a significant region×hemisphere×group interaction (*F*_2.72,269.23_ = 5.216, η^2^ = .050, *p* = .002). Follow-up comparisons showed that the FCG had greater power than the CAUG over the central region (C3 and C4) with this effect being greatest over the left hemisphere (C3). No significant main effects or interactions involving group were found for the beta band. Means and standard deviations are presented for each band in Table 1.

**Table 1:** Mean scalp EEG power for theta, alpha, and beta bands for the intervention sample.

	Left Hemisphere					Right Hemisphere				
	Frontal	Central	Parietal	Occipital	Temporal	Frontal	Central	Parietal	Occipital	Temporal
Theta (4–6 Hz)										
CAUG	3.57	3.33	3.27	2.97	3.04	3.58	3.34	3.22	2.98	2.69
	(0.33)	(0.32)	(0.35)	(0.40)	(0.54)	(0.29)	(0.31)	(0.33)	(0.41)	(0.28)
FCG	3.62	3.39	3.33	2.99	2.86	3.61	3.38	3.32	2.97	2.71
	(0.29)	(0.32)	(0.34)	(0.38)	(0.42)	(0.31)	(0.30)	(0.35)	(0.38)	(0.26)
Alpha (7–12 Hz)										
CAUG	3.27	3.28	3.13	2.94	2.71	3.27	3.30	3.15	2.92	2.45
	(0.37)	(0.49)	(0.46)	(0.52)	(0.49)	(0.36)	(0.49)	(0.48)	(0.53)	(0.35)
FCG	3.40	3.54	3.29	3.00	2.65	3.39	3.48	3.31	3.01	2.58
	(0.39)	(0.60)	(0.47)	(0.46)	(0.48)	(0.39)	(0.57)	(0.46)	(0.44)	(0.38)
Beta (13–20 Hz)										
CAUG	2.34	2.11	2.17	2.50	2.60	2.42	2.15	2.16	2.37	2.55
	(0.37)	(0.35)	(0.36)	(0.69)	(0.75)	(0.38)	(0.37)	(0.37)	(0.68)	(0.65)
FCG	2.46	2.25	2.28	2.51	2.55	2.49	2.25	2.26	2.49	2.59
	(0.41)	(0.43)	(0.42)	(0.70)	(0.60)	(0.44)	(0.45)	(0.41)	(0.69)	(0.71)

Care as usual group (CAUG; *N* = 48); Foster care group (FCG; *N* = 53); M (S.D.).

Additionally, we examined whether the intervention differentially affected boys and girls on any of the EEG measures. No interactions involving the intervention groups and gender were observed for the theta, alpha, or beta bands. Analyses were also conducted for the relative power of each band, however, no significant main effects or interactions involving our intervention groups were found.

### Timing Effects

We next examined the effects of timing-of-placement into foster care only for those receiving the BEIP intervention. To determine if power in the three EEG bands was related to the age of the child at placement into foster care, we correlated average power across all sites with age at placement. No significant correlations were found for the theta (*r*(53) = −.210, *ns*) or beta (*r*(53) = −.105, *ns*) bands; there was, however, a significant correlation between age at placement and alpha band power (*r*(53) = −.354, *p* = .009). To explore this further, we adopted an approach previously described by Nelson and colleagues [12] in which we split the FCG group into those placed before 18 months, those placed between 18 and 24 months, and those placed after 24 months of age (*n* = 11, *n* = 14, and *n* = 28, respectively). Independent samples *t*-tests were used to examine differences between the groups. Children placed before 18 months (*M* = 3.371, *SD* = .365) and between 18 and 24 months (*M* = 3.285, *SD* = .469) had significantly greater alpha power than children placed after 24 months of age (*M* = 3.023, *SD* = .318; *t*(37) = 2.949, *p* = .005 and *t*(40) = 2.134, *p* = .039, respectively). There were no differences between the groups of children placed before 18 months and those placed between 18 and 24 months (*t*(23) = .503, *p* = .620).

**Figure 1:**
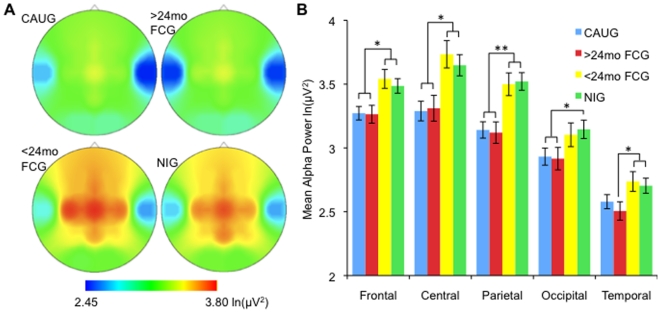
EEG scalp distribution of alpha power. (a) Scalp topography of alpha power demonstrating the timing effects for care-as-usual group (CAUG; *N* = 48), foster care group placed after 24-months (>24mo FCG; *N* = 28), foster care group placed before 24-months (<24mo FCG; *N* = 25), and the never-institutionalized (NIG; *N* = 42) group. (b) Mean alpha power across the sites for each group (* *p*<.05; *** *p*<.005).

Finally, we examined how EEG alpha power in the FCG (split by age of placement) compared to the CAUG and the NIG. Again, we found a significant effect of group (*F*_3, 139_ = 6.467, η^2^ = .122, *p*<.001) qualified by region×group and region×hemisphere×group interactions (*F*_8.66,401.19_ = 1.975, η^2^ = .041, *p* = .043 and *F*_8.66,401.39_ = 3.099, η^2^ = .063, *p* = .002, respectively). Post hoc examination of the three-way interaction revealed significant differences in alpha power between the NIG and FCG placed before 24 months at frontal, central, and parietal scalp locations compared to the CAUG and FCG placed after 24 months of age. These differences were greatest over the left hemisphere (Fig. 1).

## Discussion

Our results demonstrate a clear effect of timing of environmental enrichment on the brain electrical activity of children who experienced severe psychosocial neglect as infants and toddlers. In particular, the intervention had its greatest effect on the EEG in institutionalized children placed into foster care before 24 months of age. By 8 years of age, children in the FCG showed patterns of brain electrical activity in the alpha band that was comparable to the NIG. These effects were dependent upon age at placement, such that children who were removed from institutional care before their second birthday showed higher alpha power over frontal, central, and parietal regions. Alpha activity in the EEG signal has long been associated with attention and alertness in infants, children, and adults [13]–[15]. Our findings are consistent with a prior study showing that six years after a randomized controlled trial of an environmental enrichment intervention in preschoolers, children receiving the intervention had reduced slow frequency EEG activity and increased high frequency alpha activity that corresponded to enhanced information processing [7].

The normalization of EEG alpha activity in children who experienced severe psychosocial deprivation suggests that intervention to ameliorate deficits in brain activity as a result of significant negative early experience is possible. Other studies of the long-term outcomes of children having adverse early experiences have shown significant “catch-up” in IQ and cognitive abilities after prolonged and intensive interventions [17]–[19]. However, the Bucharest Early Intervention Project is the first study to examine the impact of the early psychosocial deprivation of institutionalization on developing brain activity. The sequence of EEG findings in this project is of particular note. The baseline assessment at around 2 years of age demonstrated group differences in the EEG, specifically higher power in the theta band and lower power in the alpha and beta bands, in children living in institutions compared to community children [1]. A follow-up assessment at 42 months of age found no effects of the intervention on the brain activity of FCG children [5]. However, at the current assessment at 8 years of age, the continued experience of an enriched environment, and the absence of psychosocial deprivation had a positive impact amongst the foster care group in terms of an important aspect of developing brain activity. Moreover, the timing effects in our findings suggest a sensitive period after which brain activity in the face of severe psychosocial deprivation is less amenable to recovery.

## Methods

### Sample

At the baseline assessment EEG was acquired on 136 infants and young children living in six institutions in Bucharest Romania. The children ranged in age from 5 to 31 months and, at the time of recruitment, had spent more than half their lives in institutional care. A never-institutionalized comparison group (NIG) of 72 children was also recruited and matched on age and gender. Following the baseline assessment, the children living in institutions were randomly assigned to receive care-as-usual (CAUG) that is, remain in their institution, or to a foster care group (FCG) who received an intervention developed by the authors. A full description of the study design can be found in [6].

The focus of the current analyses are on data collected on the follow-up assessment when the children were 8-years-old. EEG was collected for 53 (28 male) children from the FCG, 48 (25 male) children in the CAUG, and 42 (25 male) children from the NIG. The three groups did not differ on gender, χ^2^(2, *N* = 143) = .600, *p* = .741; or on age at the time of their assessment (*M* = 103.00 months, *SD* = 4.05; *F*(2,140) = 1.651, *p* = .167). An additional 3 CAUG, 2 FCG, and 12 NIG children were seen for the 8-year assessment but refused the EEG portion of the study. 17 CAUG and 13 FCG children from the original sample were unavailable for testing.

### Ethical Considerations

The University of Maryland Institutional Review Board (IRB), the Children's Hospital Boston IRB, and the Tulane University IRB approved all procedures. Written informed consent was obtained from each of the 6 local Commissions for Child Protection in Bucharest and/or the biological parents when possible (for detailed discussions of the ethical considerations see [20]–[22]). Of particular note are three policies that were put in place at the onset of the study: (1) a policy of noninterference was implemented, meaning that children in the CAUG and FCG could be placed into alternative care arrangements (e.g., government foster care, adoption, or reintegration with their biological family); (2) the noninterference policy was qualified by the stipulation that no child placed in the FCG could be returned to institutional care; and (3) an agreement with the Romanian government and a local non-governmental organization (SERA Romania) was made so that upon completion of the study, the foster families from the BEIP would continue to be supported.

### EEG Recording

EEG was recorded from 12 electrode sites (F3, F4, Fz, C3, C4, P3, P4, Pz, O1, O2, T7, and T8) and the left and right mastoids sewn in a lycra Electro-Cap (Electro-Cap International Inc., Eaton, OH) according to the international 10–20 system. EEG was collected referenced to Cz, and AFz served as ground. All electrode impedances were kept below 10kΩ. Vertical electrooculogram (EOG) was recorded using tin electrodes placed above and below the left eye to record blinks and other eye movement. The EEG and EOG signals were amplified with a gain of 5000 and 2500, respectively, and band-pass filtered from 0.1 to 100 Hz using custom bioamplifiers from James Long Company (Caroga Lake, NY). Data were digitized at 512Hz onto a PC with a 12-bit A/D converter (±2.5 V input range) and Snap-Master acquisition software (HEM Data Corporation, Southfield, MI). Before each EEG recording session, a 50µV 10Hz signal was input into all recording channels and recorded for calibration purposes.

### Procedure and EEG Analysis

EEG was recorded while the children sat quietly in a chair, alternating one-minute epochs of eyes open and eyes closed for a total of six minutes. The recorded EEG was processed using the EEG Analysis System from James Long Company. The EEG was re-referenced through software to an average mastoids reference. Epochs containing blink artifact were regressed from the EEG signals and any epochs in which the EEG signal exceeded ±200µV were excluded from further analyses. The blink-regressed and artifact scored data were spectrally analyzed using a discrete Fourier transform (DFT) with a 1-second Hanning window and 50% overlap. The three groups did not differ on number of artifact-free windows included in the analyses (*M* = 345.69, *SD* = 39.90; *F*(2,140) = 1.167, *p* = .314). Spectral power (µV^2^) was computed for the following bands: theta (4–6Hz), alpha (7–12Hz), and beta (13–20Hz). For each band absolute power was computed by taking the natural logarithm of power, similar to the procedures at the baseline and 42-month follow-up assessment [1], [5].

### Statistical Analysis

Group differences in EEG power were examined with repeated measures analyses of variance (RM-ANOVAs). Analyses were run separately for each frequency band with region (frontal, central, parietal, occipital, temporal) and hemisphere (left, right) as within-subjects factors and group as the between-subjects factor. The Greenhouse-Geisser correction was used for violations of sphericity. Analyses were run separately excluding outliers and results were similar, therefore we included all participants who provided data. Interactions not involving group are not reported.
